# Intrathoracic side‐to‐side esophagogastrostomy with a linear stapler and barbed suture in robot‐assisted Ivor Lewis esophagectomy

**DOI:** 10.1002/jso.25698

**Published:** 2019-09-18

**Authors:** Fuqiang Wang, Hanlu Zhang, Yu Zheng, Zihao Wang, Yingcai Geng, Yun Wang

**Affiliations:** ^1^ Department of Thoracic Surgery, West China Hospital Sichuan University Chengdu Sichuan China

**Keywords:** da Vinci, esophageal cancer surgery, esophagectomy, robotic surgery

## Abstract

**Background:**

The side‐to‐side anastomosis was considered a promising approach to create an intrathoracic esophagogastrostomy in the minimally invasive esophagectomy, with advantages over the side‐to‐end anastomosis with aspects of no need for additional mini‐thoracotomy and lower occurrence of stenosis. The hand‐sewing anterior aspect of the anastomosis is technically challenging in the thoracoscopic Ivor Lewis esophagectomy. Here we introduced our initial experience to facilitate this approach by using the surgical robot and barbed suture.

**Methods:**

A retrospective study of all patients underwent robot‐assisted Ivor Lewis esophagectomy with side‐to‐side esophagogastrostomy from February 2016 to September 2018 was performed. The technical details are described in this paper.

**Results:**

A total of 37 patients (35 male and 2 female, median age of 62.7 years) were successfully treated with completely robot‐assisted Ivor Lewis esophagectomy. The median total surgical time was 340 minutes including 65 minutes to perform the anastomosis. Median estimated blood loss was 120 mL and the length of hospital stay was 10 days. There was no 90‐day mortality. Three patients suffered anastomotic leakage (8.1%,3/37), who were successfully treated without reoperation.

**Conclusion:**

Our initial results imply that it is technically feasible to perform intrathoracic gastroesophageal anastomosis by taking advantage of a robotic system and knotless suturing.

## 1 INTRODUCTION

Being considered associated with reduced postoperative morbidity, the minimally invasive esophagectomy (MIE) has been widely adopted for patients with esophageal carcinoma in recent decades.^1^ However, thoracoscopic minimally invasive esophagectomy has some identified weakness in the aspect of manipulation capabilities, which become troublesome when the procedure requires fine motions such as performing anastomosis via an Ivor Lewis approach.^2^

The robotic system can offer high‐definition 3‐d vision, tremor filtration and a 7‐degree articulation of the instruments, which appears useful for precise manifestation in tiny spaces.^3^, ^4^ Taking advantage of these features, surgical robots seems to become the preferred surgical instrument in esophagogastric anastomosis by the means of hand‐sewn, circular stapled, or linear stapled approaches.^4^, ^5^, ^6^, ^7^, ^8^, ^9^

Whereas, owing to the 3‐d vision and articulated instruments, the surgical robot may provide an alternative with which to overcome these problems. Since February 2016, we have been performing minimally invasive Ivor Lewis esophagectomy (MILE) with the da Vince Si System. Here, we introduce our initial experience of intrathoracic side‐to‐side esophagogastrostomy with a linear stapler and report the short‐term outcomes.

## 2 MATERIALS AND METHODS

### 2.1 Data collection

During February 2016 to September 2018, a total of 37 patients diagnosed with esophageal carcinoma in the lower third and gastroesophageal junction underwent robot‐assisted Ivor‐Lewis esophagectomy by one medical team experienced in both minimally invasive esophagectomy and robotic surgeries. The patient demographics and perioperative parameters were collected from the institutional database, and the short‐term follow‐up was completed by return visits or telephone calls. Descriptive statistics for continuous variables are reported as median (range) and categoric variable as percentage and frequency. The study protocol was approved by the applicable institution review board of West China Hospital, Sichuan University. Written informed consents were obtained from all 37 patients.

### 2.2 Technical details

The operation was performed in two stages during the same period of anesthesia using a single‐lumen endotracheal tube. First, in the reverse Trendelenburg position, the stomach mobilization and lymph nodes dissection were completed with a four‐arm robotic platform (da Vinci Si robotic system, Intuitive Surgical, Inc.) as previously reported.^10^ After stomach mobilization, a 4‐cm wide gastric tube was fashioned extracorporeally through a 7‐cm incision in the upper abdomen. Gastric content was emptied and the gastric cavity was three times‐cleaned with diluted povidone‐iodine.

**Figure 1:**
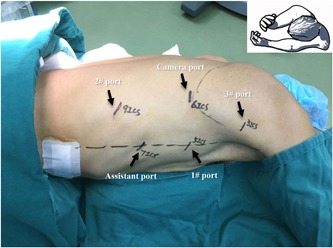
Patient positioning and port placement for thoracic phase. Patient position: left lateral decubitus position, tilted 45° toward the prone position. Camera port: 6th ICS posterior to scapula angle. Port for robotic arm 1: 5th ICS anterior to the scapular rim. Port for robotic arm 2: 9th ICS posterior to the posterior axillary line. Port for robotic arm 3: 3rd ICS anterior to the scapula. Assistant port: 7th ICS anterior to the posterior axillary line [Color figure can be viewed at wileyonlinelibrary.com]

In the second stage, the patient was repositioned in a left semi‐prone position. Four robotic ports and one assistant port are placed as shown in Figure 1. The 12‐mm camera port was placed at the sixth intercostal space (ICS) posterior to scapula angle. Three 8‐mm trocars for robotic instruments were inserted as follows: one for arm 3 at the third ICS anterior to the scapula, one for arm 2 at the ninth ICS posterior to the posterior axillary line, and the third for arm 1 at the fifth ICS anterior to the scapular rim. The 12‐mm assistant port was located at the seventh ICS anterior to the posterior axillary line.

**Figure 2:**
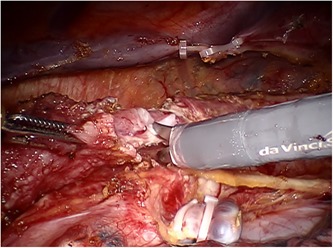
Transecting the esophagus at the level of the azygos arch. The mucosal and muscular layers are transected separately, and adequate mucosa is retained to countervail its retraction [Color figure can be viewed at wileyonlinelibrary.com]

**Figure 3:**
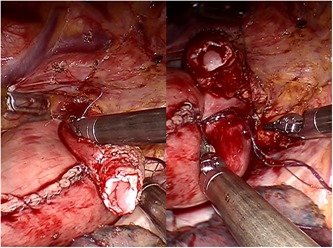
Two stay sutures are placed to keep the gastric tip aligned with the esophageal wall at the apex of the posterior mediastinum [Color figure can be viewed at wileyonlinelibrary.com]

**Figure 4:**
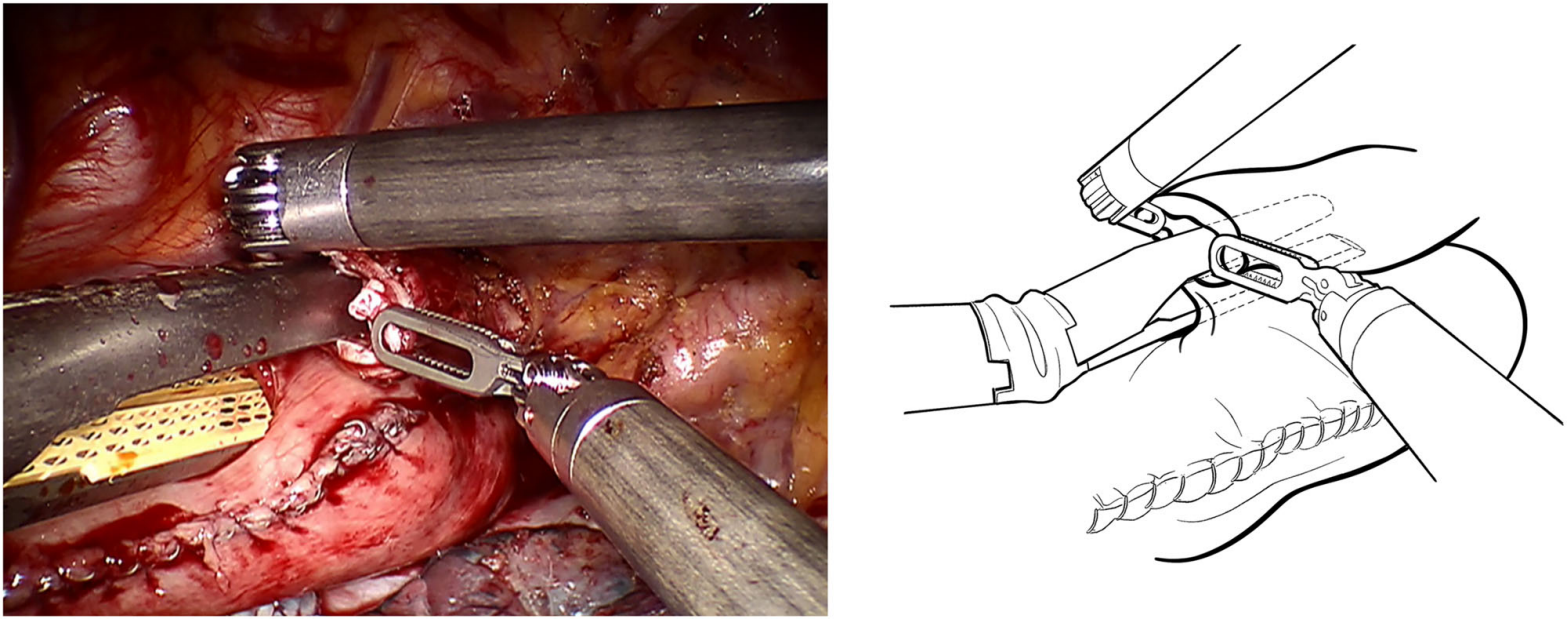
The linear stapler is introduced through the trocar for robotic arm 2, with two jaws inserting in gastric tube and esophagus separately [Color figure can be viewed at wileyonlinelibrary.com]

**Figure 5:**
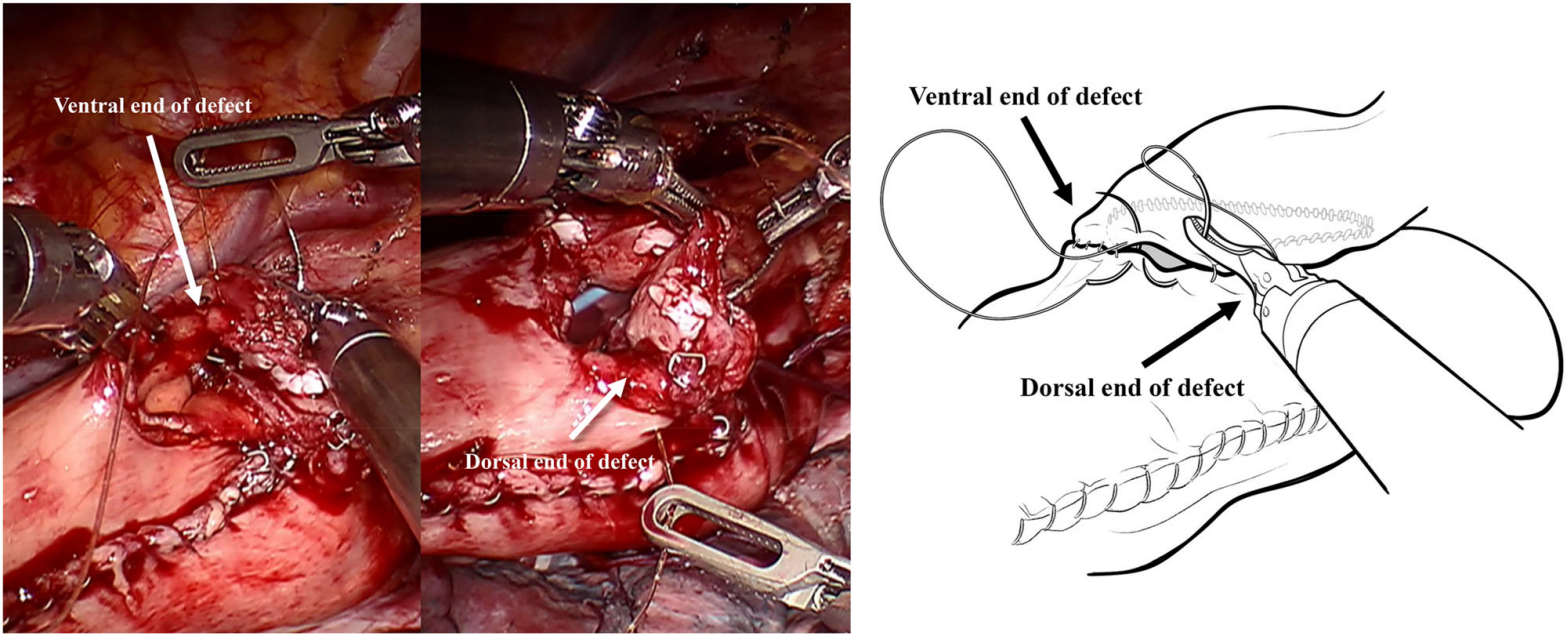
Two self‐locking barbed sutures are placed at each end of the remaining defect and close the defect of the whole layer by running suture from end to middle [Color figure can be viewed at wileyonlinelibrary.com]

**Figure 6:**
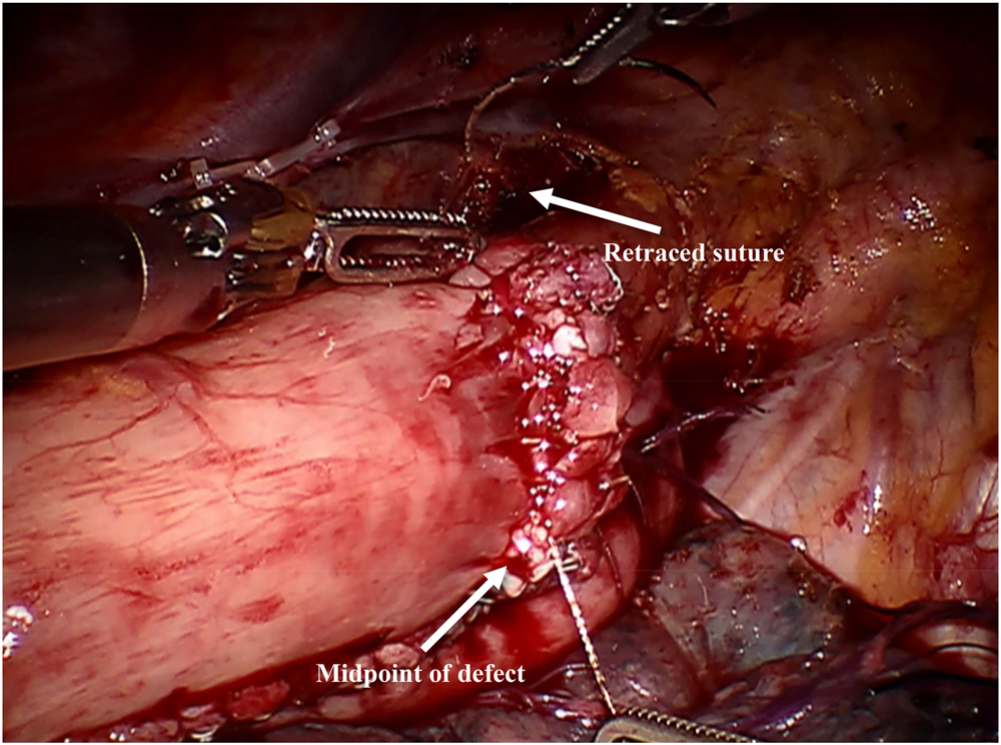
After cross over at middle of defect, the running sutures retrace to their own ends to embed the anastomosis [Color figure can be viewed at wileyonlinelibrary.com]

After the circumferential mobilization of thoracic esophagus and dissection of paraoesophageal tissue, we start to fashion anastomosis by transecting the thoracic esophagus at the level of the azygos arch with the monopolar curved scissors (Figure 2). The gastric conduit was then delivered into the thoracic cavity just beneath the esophageal stump, and the tip of the gastric tube was stitched to the esophageal wall at the top of hemithorax subsequently (Figure 3). After making a 1‐cm hole in the upper side of the gastric tube, robotic arm 2 was temporarily undocked. Then through this port, the linear stapler (Echelon Flex Powered Stapler 60 mm, Ethicon Endosurgery) was introduced into the orifice of the gastric tube and esophageal stump, and a 4‐cm long side‐to‐side stapling procedure applied to form the posterior aspect of the anastomosis (Figure 4). Two self‐locking barbed sutures (Stratafix Sporal 3/0, Ethicon Endosurgery) were placed at each end of the remaining defect, and we closed the defect of the whole layer by running suture from the end to the middle (Figure 5). After cross over at the middle, the running sutures were retraced to their own ends to embed the anastomosis (Figure 6).

Hemostasis was checked, and the right hemithorax was irrigated with normal saline. A 28‐F chest tube was inserted into the thoracic cavity through the camera port, and an 18‐F silicone tube was placed in postmediastinum behind the gastric conduit through the port for robotic arm 2.

### 2.3 Postoperative management

Total parenteral nutrition was used in the first 4 days after operation and the oral intake began at postoperative day (POD) 3 to POD5 with a liquid diet, to which was added Methylene blue to explore potential anastomotic fistula. With the absence of leakage, the drainage tube would be removed at POD7 to POD10. All patients were discharged once oral feeding could be tolerated.

## 3 RESULTS

The demographic, intra‐ and peri‐operative data of all 37 patients are listed (Table 1). In summary, the median age of patients was 62.7 years and the median body mass index (BMI) was 22.1 kg/m^2^. A total of eight patients with local advanced esophageal cancer underwent neoadjuvant therapy in this case. The median total surgical time was 340 minutes, with 165 minutes for the thoracic phase and 65 minutes required to create anastomosis. The estimated blood loss ranged from 50 mL to 160 mL, with a median of 120 mL. The median length of hospital stay was 10 days. Postoperative pathology shows that there were 26 cases of squamous carcinoma, nine cases of adenocarcinoma, one case of neuroendocrine carcinoma of and one case of small‐cell carcinoma. All patients had negative margins. Postoperative complications were classified according to Esophagectomy Complications Consensus Group (ECCG) guidelines.^11^

**Table 1:** Patients’ characteristics and outcomes

Patients’ characteristics	All patients (*n* = 37)	%
Age,y	62.7 (range, 46‐75)	
Sex		
male	35	94.6
female	2	5.4
BMI, kg/m^2^	22.1 (range, 15.1‐29.4)	
Comorbidities		
Chronic gastritis	14	37.8
Duodenitis	2	5.4
Hypertension	4	10.8
Emphysema	4	10.8
COPD	5	13.5
Arrhythmia	2	5.4
Zenker's Diverticulum	1	2.7
Neoadjuvant therapy	8	21.6
Histology		
Squamous carcinoma	26	70.3
Adenocarcinoma	9	24.3
Neuroendocrine carcinoma	1	2.7
Small‐cell carcinoma	1	2.7
Surgical Time		
Total, min	340(range, 300‐475)	
Port step and docking, min	20(range,15‐32)	
Intrathoracic process, min	165 (range, 140‐275)	
Time for Anastomosis, min	65(range, 40‐90)	
Bleeding volume, mL	120 (range, 50‐160)	
Length of hospital stay	10 (range, 9‐25)	
Postoperative complication^a^		
Anastomotic leakage	3	8.1
Hoarseness	2	5.4
Chylothorax	1	2.7
Pneumonia^b^	3	8.1
Atrial fibrillation	1	2.7
Phlebothrombosis	2	5.4
Pathological stage		
IA	2	5.4
IB	2	5.4
IIA	8	21.6
IIB	13	35.1
IIIA	3	8.1
IIIB	6	16.2
IIIC	3	8.1

Abbreviations: BMI, body mass index; COPD, chronic obstructive pulmonary disease.

^a^ postoperative complication was classified according to Esophagectomy Complications Consensus Group.

^b^ pneumonia was defined according to the American Thoracic Society and Infectious Diseases Society of America.

A total of three patients suffered postoperative anastomotic leakage. All three fistulas were developed at the anastomosed area, and no airway fistula or enterocutaneous fistula occurred in this series. Anastomotic fistula in two patients was confirmed by methylene blue which was drained from the thoracic cavity and mediastinum. The other anastomotic fistula was diagnosed by digital gastrointestinal radiography. All three patients were successfully treated by drainage and/or irrigation without reoperation.

During the follow‐up period for 3 to 16 months, stenosis occurred during the 16th month after discharge in one patient, and another patient died at the 12th month postoperatively as a result of neoplasm recurrence.

## 4 COMMENT

Considering its advantages including low stricture rates and absence of need for an extra minithoracotomy to insert the stapler, the side‐to‐side anastomosis has been considered a promising approach in the MILE. However, technical difficulties in endoscopic suturing may hinder the extensive adoption of this procedure. The application of a surgical robot may find a way out of this dilemma by significantly improving the surgeon's ability to suture even in deep and narrow anatomical sites such as the posterior mediastinum. Actually, the robot‐assisted suturing was so facilitating that a hand‐sewing anastomosis was achievable even in the posterior aspect of the anastomosis for low tension in anastomosis.^9^ However, according to our own experience, both exposure and operation condition are more unsatisfactory in the posterior aspect when comparing with the anterior one. We agree with Dr. Cerfolio to staple instead of hand‐sew the posterior aspect.^9^ We believe that it is an acceptable compromise to create an anastomosis using a hybrid technique involving stapling the posterior wall and hand‐sewing the anterior wall.

Triangular stapling is another anastomotic technique totally using linear staplers, which is reported being associated with a decreased rate of anastomotic complications.^12^ Nevertheless, the gastric conduit and remnant esophagus need to be stapled three times in three different directions in the classic triangulating stapling technique, which is a technical challenge for intrathoracic manipulation in MIE.^13^, ^14^, ^15^ We have several lessons when performing triangular stapling anastomosis thoracoscopically. Thus, we were not attempting triangular stapling technique in robotic intrathoracic esophagogastrostomy.

The knotless barbed suture appears to be ideal for endoscopic suturing according to the literature and our own experience.^6^, ^7^, ^8^ First, it is self‐retaining, thus liberating the surgeon from the need to hold the suture. Second, it provides knotless wound closure, which reduces the time to make knots. Furthermore, the barbed suture allows multiple points of fixation along with the closure, compared with only two points of fixation at the knots with traditional suturing. This should permit a greater distribution of tensile strength along the wound and increase the surface area of adhesion between tissues.

Although creating a gastric conduit intracorporeally would cause less trauma in the abdominal phase of robot‐assisted Ivor‐Lewis esophagectomy, we still chose to complete it extracorporeally through a 7‐cm incision in the upper abdomen. Therefore, we irrigated the gastral cavity before anastomosing. Due to the barrier resulting from a mass of esophageal carcinoma, sometimes the nasogastric tube cannot be placed in the proper location. This led to a poor preoperative preparation where a mass of content remained in the gastral cavity. Under such circumstance, gastric content would leak from the orifice of the gastric tube to the abdominal cavity during anastomosis, which is one of the major sources of intrathoracic abscess and postoperative contamination in previous years in our center. Since adoption of irrigation before anastomosis, we recorded a decrease in postoperative intrathoracic abscess.

After creating the posterior part of anastomosis with linear stapler, we stitched the anterior defect from two corners and combined at the midpoint, which was different from the typical end‐to‐end modus operandi. During operation, we found that the surgical viewing became worse from the midline to the corner, and this problem was aggravated when sewing up the corner. We speculated that starting at each corner and finishing the suturing at the midline could ameliorate this problem.

There are three postoperative fistulas in our cohort: one presumed cause is the compromised blood perfusion associated with the longer esophageal stump in the side‐to‐side approach compared with hand‐sewn or end‐to‐side anastomosis. Hodari^16^ used real‐time perfusion assessment to ensure anastomosis is placed in well‐perfused parts of esophagus in robot‐assisted minimally invasive Ivor Lewis esophagectomy. With such technique of perfusion assessment, a total of three patients (5.5%, 3/54) experienced anastomotic leak in the study of Dr. Hodari.

This study contained several limitations: first, due to the limitations of retrospective study, generalizations of several results were subject to potential bias; second, only 37 patients were included in this study, therefore, a prospective study with large sample size is recommended. Notwithstanding these limitations, we believe that, with such advantages of robotic surgery, clinical outcomes of robotic Ivor Lewis esophagectomy can be further promoted after completion of the learning curve.

In summary, the utilization of surgical robot and barbed suture could facilitate the creation of intrathoracic anastomosis in MILE. It seems to be a promising alternative to open or traditional thoracoscopic surgery.

## DATA AVAILABILITY STATEMENT

The data that support the findings of this study are available on request from the corresponding author. The data are not publicly available due to privacy or ethical restrictions.

## SYNOPSIS

The technical difficulties may hinder the extensive adoption of the side‐to‐side anastomosis in the minimally invasive Ivor Lewis esophagectomy. Our initial results imply it is technically feasible to perform intrathoracic gastroesophageal anastomosis by taking advantage of the robotic system and knotless suture.
